# 2-Acetyl­anilinium chloride

**DOI:** 10.1107/S1600536811004739

**Published:** 2011-02-12

**Authors:** R. Prasath, P. Bhavana, Seik Weng Ng, Edward R. T. Tiekink

**Affiliations:** a Department of Chemistry, BITS, Pilani – K. K. Birla Goa Campus, Goa 403 726, India; b Department of Chemistry, University of Malaya, 50603 Kuala Lumpur, Malaysia

## Abstract

The cation of the title salt, C_8_H_10_NO^+^·Cl^−^, is essentially planar [C—C—C—C torsion angle = 4.6 (2)°], the conformation being stabilized by an intra­molecular N—H⋯O hydrogen bond. In the crystal, centrosymmetric aggregates are formed *via* N—H⋯Cl hydrogen bonds. These dimeric aggregates are sustained in the crystal packing by a combination of C—H⋯Cl, C—H⋯O and C—O⋯π [O⋯ring centroid(benzene ring) = 3.1871 (13) and 3.3787 (13) Å] inter­actions.

## Related literature

For background to structural studies of quinoline derivatives, see: Kaiser *et al.* (2009 ▶).
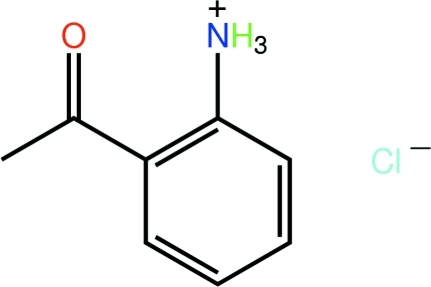


## Experimental

### 

#### Crystal data

C_8_H_10_NO^+^·Cl^−^
*M* *_r_* = 171.62
Monoclinic, 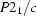

*a* = 4.8979 (1) Å
*b* = 15.8136 (4) Å
*c* = 10.8203 (3) Å
β = 102.569 (3)°
*V* = 817.98 (3) Å^3^
*Z* = 4
Mo *K*α radiation
μ = 0.41 mm^−1^
*T* = 100 K
0.30 × 0.25 × 0.20 mm

#### Data collection

Agilent Supernova Dual diffractometer with an Atlas detector
Absorption correction: multi-scan (*CrysAlis PRO*; Agilent, 2010 ▶) *T*_min_ = 0.920, *T*_max_ = 1.000
3262 measured reflections
1436 independent reflections
1256 reflections with *I* > 2σ(*I*)
*R*_int_ = 0.019

#### Refinement

*R*[*F* ^2^ > 2σ(*F* ^2^)] = 0.029
*wR*(*F* ^2^) = 0.080
*S* = 1.07
1436 reflections
113 parameters
H atoms treated by a mixture of independent and constrained refinement
Δρ_max_ = 0.20 e Å^−3^
Δρ_min_ = −0.27 e Å^−3^

### 

Data collection: *CrysAlis PRO* (Agilent, 2010 ▶); cell refinement: *CrysAlis PRO*; data reduction: *CrysAlis PRO*; program(s) used to solve structure: *SHELXS97* (Sheldrick, 2008 ▶); program(s) used to refine structure: *SHELXL97* (Sheldrick, 2008 ▶); molecular graphics: *ORTEP-3* (Farrugia, 1997 ▶) and *DIAMOND* (Brandenburg, 2006 ▶); software used to prepare material for publication: *publCIF* (Westrip, 2010 ▶).

## Supplementary Material

Crystal structure: contains datablocks global, I. DOI: 10.1107/S1600536811004739/hg2797sup1.cif

Structure factors: contains datablocks I. DOI: 10.1107/S1600536811004739/hg2797Isup2.hkl

Additional supplementary materials:  crystallographic information; 3D view; checkCIF report

## Figures and Tables

**Table 1:** Hydrogen-bond geometry (Å, °)

*D*—H⋯*A*	*D*—H	H⋯*A*	*D*⋯*A*	*D*—H⋯*A*
N1—H1n⋯Cl1	0.95 (2)	2.23 (2)	3.1463 (15)	163 (2)
N1—H2n⋯Cl1^i^	0.92 (2)	2.24 (2)	3.1366 (15)	165 (2)
N1—H3n⋯O1	0.87 (2)	1.95 (2)	2.6778 (18)	140 (2)
C5—H5⋯Cl1^ii^	0.95	2.73	3.5332 (18)	142
C6—H6⋯O1^iii^	0.95	2.59	3.2496 (19)	127

Symmetry codes: (i) 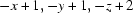; (ii) 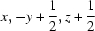; (iii) 
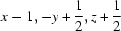
.

**Fig. 1.**
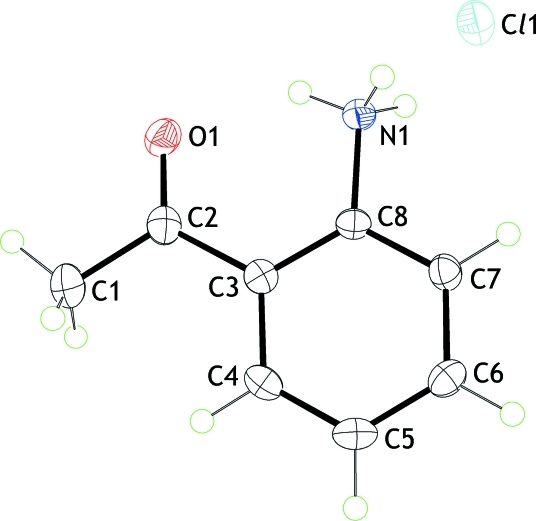
The constituent ions of salt (I) showing the atom-labelling scheme and displacement ellipsoids at the 50% probability level.

**Fig. 2.**
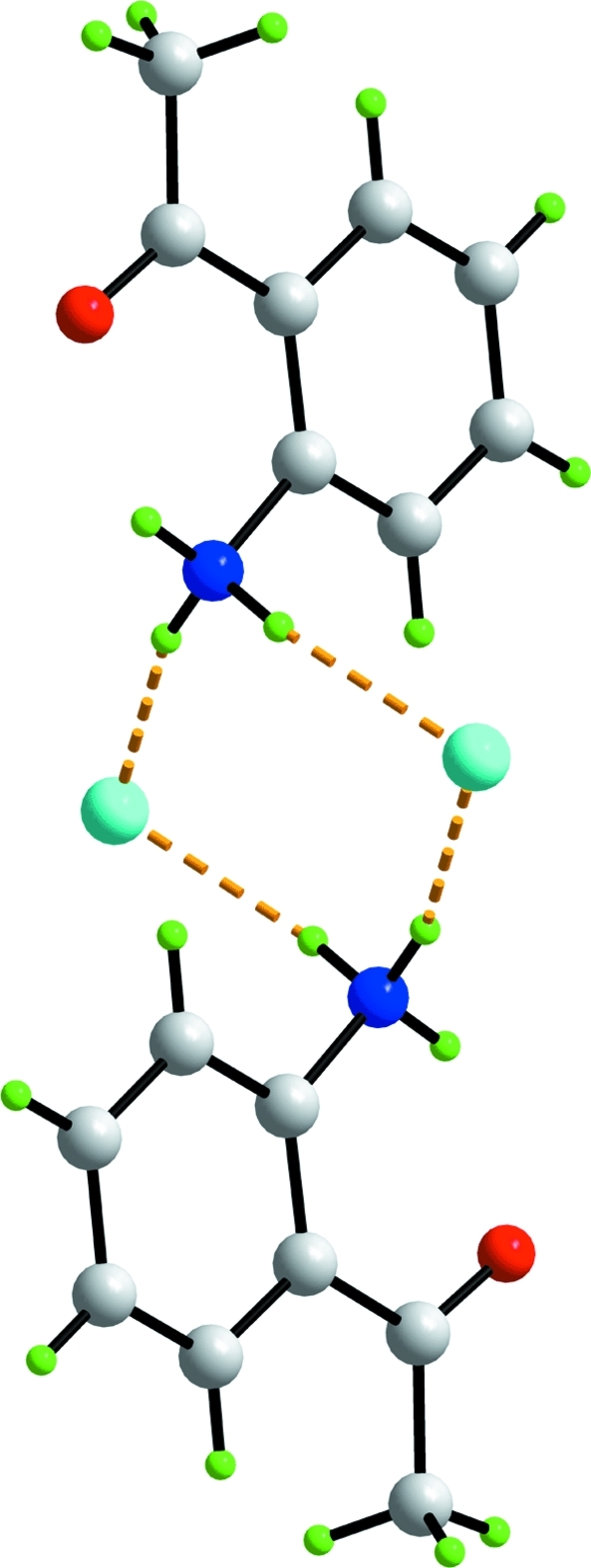
Supramolecular dimer in (I) mediated by N–H···Cl hydrogen bonding, shown as orange dashed lines.

**Fig. 3.**
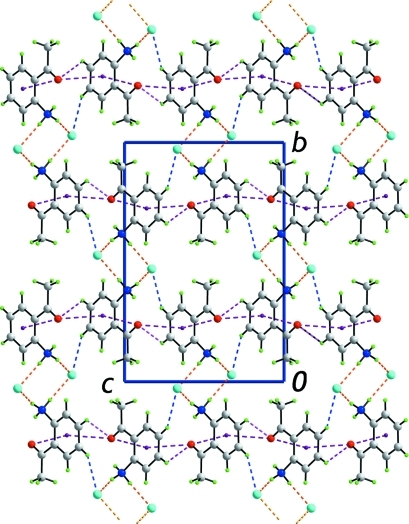
Unit-cell contents shown in projection down the a axis in (I). The N–H···Cl hydrogen bonding, and C–H···Cl, C–H···O and C–O···π contacts are shown as orange, blue, pink, and purple dashed lines, respectively.

## supplementary crystallographic information

### Comment

The title salt, (I), was obtained as an unexpected product during the attempted synthesis of a quinoline derivative, investigated as a part of an on-going programme into the synthesis and structural chemistry of quinolines of interest owing to their putative anti-malarial activity (Kaiser *et al.*, 2009).

Ionic (I), Fig. 1, comprises a 2-acetylanilinium cation and a chloride anion. The cation is essentially planar with the acetyl group only slightly twisted out of the plane of the benzene ring to which it is connected, the C1–C2–C3–C4 torsion angle = 4.6 (2) °. The observed conformation is stabilized by an intramolecular N–H···O hydrogen bond, Table 1.

In the crystal packing, centrosymmetrically related molecules are connected into a supramolecular dimer *via* N–H···Cl hydrogen bonds, Table 1 and Fig. 2. The dimeric aggregates are arranged into layers in the *ac* plane *via* a combination of C–H···O, Table 1, and C—O···π contacts [C2–O1···*Cg*(C3—C8)^i^ = 3.1871 (13) Å with the angle at O1 = 96.44 (10) ° for *i*: 1 + *x*, *y*, *z*; and C2–O1···*Cg*(C3—C8)^ii^ = 3.3787 (13) Å with the angle at O1 = 98.36 (9) ° for *ii*: *x*, 1/2 - *y*, -1/2 + *z*]. The presence of C–H···Cl interactions, Table 1, contributes to the stability of the structure along the *b* axis, Fig. 3.

### Experimental

A mixture of 2-aminoacetophenone (0.01 *M*), acetophenone (0.01 *M*) and a catalytic amount of conc. HCl was heated on a water bath for 10 min. The resultant solid was filtered, dried and purified by column chromatography using a 1:5 mixture of ethyl acetate and hexane. Re-crystallization was by slow evaporation of an acetone solution of (I) which yielded colourless needles. *M*.pt. 421–423 K. Yield: 66%. The X-ray study showed that the original 2-aminoacetophenone had been protonated and crystallized as a chloride salt.

### Refinement

Carbon-bound H-atoms were placed in calculated positions (C—H 0.95 to 0.98 Å) and were included in the refinement in the riding model approximation, with *U*_iso_(H) set to 1.2 to 1.5*U*_equiv_(C). The N-bound H-atoms were located in a difference Fourier map and refined freely.

### Crystal data

**Table:**

C_8_H_10_NO^+^·Cl^−^	*F*(000) = 360
*M**_r_* = 171.62	*D*_x_ = 1.394 Mg m^−^^3^
Monoclinic, *P*2_1_/*c*	Mo *K*α radiation, λ = 0.71073 Å
Hall symbol: -P 2ybc	Cell parameters from 2016 reflections
*a* = 4.8979 (1) Å	θ = 2.3–29.1°
*b* = 15.8136 (4) Å	µ = 0.41 mm^−^^1^
*c* = 10.8203 (3) Å	*T* = 100 K
β = 102.569 (3)°	Pris,, colourless
*V* = 817.98 (3) Å^3^	0.30 × 0.25 × 0.20 mm
*Z* = 4	

### Data collection

**Table:**

Agilent Supernova Dual diffractometer with an Atlas detector	1436 independent reflections
Radiation source: SuperNova (Mo) X-ray Source	1256 reflections with *I* > 2σ(*I*)
Mirror	*R*_int_ = 0.019
Detector resolution: 10.4041 pixels mm^-1^	θ_max_ = 25.0°, θ_min_ = 2.3°
ω scans	*h* = −5→5
Absorption correction: multi-scan (*CrysAlis PRO*; Agilent, 2010)	*k* = −18→17
*T*_min_ = 0.920, *T*_max_ = 1.000	*l* = −10→12
3262 measured reflections	

### Refinement

**Table:**

Refinement on *F*^2^	Primary atom site location: structure-invariant direct methods
Least-squares matrix: full	Secondary atom site location: difference Fourier map
*R*[*F*^2^ > 2σ(*F*^2^)] = 0.029	Hydrogen site location: inferred from neighbouring sites
*wR*(*F*^2^) = 0.080	H atoms treated by a mixture of independent and constrained refinement
*S* = 1.07	*w* = 1/[σ^2^(*F*_o_^2^) + (0.0393*P*)^2^ + 0.2093*P*] where *P* = (*F*_o_^2^ + 2*F*_c_^2^)/3
1436 reflections	(Δ/σ)_max_ = 0.001
113 parameters	Δρ_max_ = 0.20 e Å^−^^3^
0 restraints	Δρ_min_ = −0.27 e Å^−^^3^

### Special details

**Table:**

Geometry. All s.u.'s (except the s.u. in the dihedral angle between two l.s. planes) are estimated using the full covariance matrix. The cell s.u.'s are taken into account individually in the estimation of s.u.'s in distances, angles and torsion angles; correlations between s.u.'s in cell parameters are only used when they are defined by crystal symmetry. An approximate (isotropic) treatment of cell s.u.'s is used for estimating s.u.'s involving l.s. planes.
Refinement. Refinement of *F*^2^ against ALL reflections. The weighted *R*-factor *wR* and goodness of fit *S* are based on *F*^2^, conventional *R*-factors *R* are based on *F*, with *F* set to zero for negative *F*^2^. The threshold expression of *F*^2^ > 2σ(*F*^2^) is used only for calculating *R*-factors(gt) *etc*. and is not relevant to the choice of reflections for refinement. *R*-factors based on *F*^2^ are statistically about twice as large as those based on *F*, and *R*- factors based on ALL data will be even larger.

### Fractional atomic coordinates and isotropic or equivalent isotropic displacement parameters (Å^2^)

**Table:**

	*x*	*y*	*z*	*U*_iso_*/*U*_eq_	
Cl1	0.10927 (9)	0.47141 (2)	0.83017 (4)	0.02647 (17)	
O1	0.8706 (2)	0.23737 (7)	0.91735 (11)	0.0277 (3)	
N1	0.6529 (3)	0.38178 (9)	0.98223 (14)	0.0210 (3)	
H1N	0.508 (4)	0.4081 (12)	0.9220 (19)	0.036 (5)*	
H2N	0.753 (4)	0.4233 (13)	1.0322 (19)	0.033 (5)*	
H3N	0.759 (4)	0.3532 (12)	0.9418 (19)	0.034 (5)*	
C1	0.7572 (5)	0.10071 (11)	0.97971 (19)	0.0377 (5)	
H1A	0.8665	0.0826	0.9186	0.057*	
H1B	0.5674	0.0776	0.9552	0.057*	
H1C	0.8466	0.0800	1.0642	0.057*	
C2	0.7435 (3)	0.19573 (10)	0.98154 (15)	0.0233 (4)	
C3	0.5701 (3)	0.23753 (10)	1.06204 (15)	0.0197 (4)	
C4	0.4392 (4)	0.18953 (10)	1.14121 (16)	0.0238 (4)	
H4	0.4639	0.1299	1.1444	0.029*	
C5	0.2751 (4)	0.22678 (11)	1.21474 (16)	0.0253 (4)	
H5	0.1875	0.1928	1.2673	0.030*	
C6	0.2381 (3)	0.31345 (10)	1.21206 (16)	0.0240 (4)	
H6	0.1249	0.3391	1.2626	0.029*	
C7	0.3663 (3)	0.36268 (10)	1.13559 (15)	0.0219 (4)	
H7	0.3423	0.4223	1.1337	0.026*	
C8	0.5289 (3)	0.32504 (10)	1.06211 (15)	0.0185 (4)	

### Atomic displacement parameters (Å^2^)

**Table:**

	*U*^11^	*U*^22^	*U*^33^	*U*^12^	*U*^13^	*U*^23^
Cl1	0.0285 (3)	0.0215 (3)	0.0298 (3)	0.00177 (17)	0.00704 (19)	−0.00224 (17)
O1	0.0280 (7)	0.0299 (7)	0.0275 (7)	0.0043 (5)	0.0113 (6)	−0.0002 (5)
N1	0.0222 (8)	0.0193 (8)	0.0235 (8)	−0.0005 (7)	0.0091 (7)	0.0013 (7)
C1	0.0527 (13)	0.0241 (10)	0.0401 (12)	0.0081 (9)	0.0182 (10)	−0.0017 (9)
C2	0.0230 (9)	0.0236 (9)	0.0212 (9)	0.0040 (7)	0.0003 (7)	−0.0008 (7)
C3	0.0173 (8)	0.0210 (9)	0.0199 (8)	0.0010 (7)	0.0016 (7)	0.0006 (7)
C4	0.0251 (9)	0.0188 (9)	0.0262 (9)	0.0001 (7)	0.0027 (7)	0.0048 (7)
C5	0.0226 (9)	0.0300 (10)	0.0238 (9)	−0.0034 (8)	0.0060 (7)	0.0067 (8)
C6	0.0196 (9)	0.0304 (10)	0.0232 (9)	0.0015 (7)	0.0073 (7)	−0.0005 (8)
C7	0.0205 (9)	0.0191 (9)	0.0264 (9)	0.0015 (7)	0.0058 (7)	−0.0005 (7)
C8	0.0158 (8)	0.0205 (8)	0.0190 (8)	−0.0017 (6)	0.0031 (7)	0.0017 (7)

### Geometric parameters (Å, °)

**Table:**

O1—C2	1.221 (2)	C3—C8	1.399 (2)
N1—C8	1.466 (2)	C3—C4	1.400 (2)
N1—H1N	0.95 (2)	C4—C5	1.380 (2)
N1—H2N	0.92 (2)	C4—H4	0.9500
N1—H3N	0.87 (2)	C5—C6	1.382 (2)
C1—C2	1.504 (2)	C5—H5	0.9500
C1—H1A	0.9800	C6—C7	1.383 (2)
C1—H1B	0.9800	C6—H6	0.9500
C1—H1C	0.9800	C7—C8	1.377 (2)
C2—C3	1.496 (2)	C7—H7	0.9500
			
C8—N1—H1N	109.3 (12)	C4—C3—C2	120.68 (15)
C8—N1—H2N	108.9 (12)	C5—C4—C3	121.54 (15)
H1N—N1—H2N	108.2 (16)	C5—C4—H4	119.2
C8—N1—H3N	110.2 (13)	C3—C4—H4	119.2
H1N—N1—H3N	108.7 (18)	C4—C5—C6	120.10 (16)
H2N—N1—H3N	111.5 (18)	C4—C5—H5	120.0
C2—C1—H1A	109.5	C6—C5—H5	120.0
C2—C1—H1B	109.5	C5—C6—C7	119.76 (16)
H1A—C1—H1B	109.5	C5—C6—H6	120.1
C2—C1—H1C	109.5	C7—C6—H6	120.1
H1A—C1—H1C	109.5	C8—C7—C6	119.82 (15)
H1B—C1—H1C	109.5	C8—C7—H7	120.1
O1—C2—C3	121.12 (15)	C6—C7—H7	120.1
O1—C2—C1	120.10 (16)	C7—C8—C3	121.98 (15)
C3—C2—C1	118.77 (15)	C7—C8—N1	116.21 (14)
C8—C3—C4	116.80 (15)	C3—C8—N1	121.80 (14)
C8—C3—C2	122.52 (14)		
			
O1—C2—C3—C8	4.3 (2)	C5—C6—C7—C8	0.3 (2)
C1—C2—C3—C8	−175.12 (16)	C6—C7—C8—C3	−0.1 (2)
O1—C2—C3—C4	−176.05 (15)	C6—C7—C8—N1	178.95 (14)
C1—C2—C3—C4	4.6 (2)	C4—C3—C8—C7	−0.4 (2)
C8—C3—C4—C5	0.7 (2)	C2—C3—C8—C7	179.28 (15)
C2—C3—C4—C5	−179.04 (15)	C4—C3—C8—N1	−179.37 (15)
C3—C4—C5—C6	−0.4 (3)	C2—C3—C8—N1	0.3 (2)
C4—C5—C6—C7	−0.1 (3)		

### Hydrogen-bond geometry (Å, °)

**Table:**

*D*—H···*A*	*D*—H	H···*A*	*D*···*A*	*D*—H···*A*
N1—H1n···Cl1	0.95 (2)	2.23 (2)	3.1463 (15)	162.9 (17)
N1—H2n···Cl1^i^	0.92 (2)	2.24 (2)	3.1366 (15)	165.0 (17)
N1—H3n···O1	0.87 (2)	1.946 (19)	2.6778 (18)	140.3 (18)
C5—H5···Cl1^ii^	0.95	2.73	3.5332 (18)	142
C6—H6···O1^iii^	0.95	2.59	3.2496 (19)	127

Symmetry codes: (i) −*x*+1, −*y*+1, −*z*+2; (ii) *x*, −*y*+1/2, *z*+1/2; (iii) *x*−1, −*y*+1/2, *z*+1/2.
